# A Worker’s Struggle

**DOI:** 10.4269/ajtmh.25-0111

**Published:** 2025-07-10

**Authors:** Sakan Binte Imran, Shadman Mahmood Khan Pathan

**Affiliations:** ^1^ Sir Salimullah Medical College Mitford Hospital, Dhaka, Bangladesh;; ^2^ Old Dominion University, Norfolk, Virginia

The casualty department at Sir Salimullah Medical College Mitford Hospital was crowded, as it always was. The air smelled of antiseptics, and hurried footsteps echoed through the narrow corridors. It was another day of internship, and I had just finished checking on a patient when a nurse called me over.

“There’s a new case in Trauma. Factory worker. Severe hand injury.”

I hurried over and found a man sitting on the examination table. His face was pale, and his forehead was glistening with sweat. His right hand was wrapped in a blood-soaked cloth. His left hand trembled as he clutched his lap.

His name was Abdul Karim. He worked in a small steel factory on the outskirts of Dhaka. The accident had happened that morning. A moment’s distraction, a slip of the hand, and the sharp blade of the machine had cut through his fingers. The factory supervisor had bandaged his hand hastily and sent him away, warning him not to make a fuss.

Karim winced as we removed the cloth. Three of his fingers were severed, leaving only raw, exposed flesh. His breath came in short gasps, not just from pain but from something deeper—the fear of what would come next.

“My hand, doctor… How will I work?” His voice cracked.

His wife stood beside him, holding back tears. She was a woman in her early thirties, wearing a faded saree, her eyes hollow with exhaustion. Next to her, their eldest son, who was barely 10 years old, clutched the hem of her saree, looking up at his father with quiet fear.

“He is the only one earning,” his wife said in a hushed voice, almost as if she were afraid of her own words. “The children… My mother-in-law… How will we survive?”

I felt a heaviness in my chest. I had seen such cases before. In the months of my internship, I had treated rickshaw pullers, construction workers, welders, and tailors, all of whom were injured in similar ways. They had suffered crushed hands and legs, severed fingers, deep burns, assaults, and head trauma, and some were even brought in dead. These injuries did not just take a piece of flesh but an entire livelihood.

After handing over the paperwork for the patient’s admission and further care to one of my fellow interns, I immediately asked the patient to undergo an X-ray, marking his ticket as urgent. His fingers had been severely injured (partially severed), but there was still a chance to save them, at least to some extent. The X-ray was crucial to assess the damage and plan the next steps.

He returned shortly with the X-ray in hand. Without wasting a moment, I escorted him to the operating room. I quickly scrubbed in, donned a gown, and draped a McIntosh over it. Calling the operating theater nurse and assistant, I requested the necessary equipment. They promptly provided everything I needed, and I additionally asked for two K-wires. The experienced operating theater assistant raised an eyebrow, grimacing slightly. “K-wires? Here?” he asked, his tone laced with skepticism.

I met his gaze firmly and replied, “Certainly. We can save at least two fingers entirely, though one might require amputation. When there’s a chance to restore functionality, why wouldn’t we take it?”

He seemed to soften at my explanation, nodding slowly. “Yes, you’re right. We should go for it,” he agreed.

After meticulously cleaning the patient’s hand and the soiled wound, I administered local anesthesia. The patient’s tearful eyes locked onto mine, filled with a mixture of fear and hope. “Can my fingers be saved?” he whispered, his voice trembling.

I placed a reassuring hand on the sterile hand portion. “We’re doing everything we can. Let’s hope for the best,” I said gently, although the weight of his anxiety was palpable.

The procedure went as planned. We successfully inserted K-wires into two of the fingers, preserving as much of their structure as possible. Unfortunately, one finger required a mid-phalangeal amputation. However, out of the three injured fingers, two were saved.

Once the procedure was completed, we carefully bandaged the wound and instructed him to apply the prescribed medications regularly. “Come back after ten days for stitch removal,” I reminded him.

As the reality of what had just happened sank in, the patient took a deep, shuddering breath. Tears streamed down his face as he began to cry, not just from pain but from relief. “I was so scared,” he admitted, his voice breaking. “I couldn’t show it in front of my family, but I kept thinking about how my entire life could have changed if my hand wasn’t saved. Everything I’ve worked for—it could have fallen apart. But today, you saved me.”

His gratitude was overwhelming. He asked for our contact details, but I explained as gently as I could that as a government hospital, we could not share personal contacts. “But you can always come back and find us here,” I assured him. “We’ll be here to help you through your recovery.”

He nodded, wiping his tears. “I’ll follow all the instructions carefully and come back in ten days,” he promised.

I added, “You’ll also need follow-up care for rehabilitation. Please make sure to stay in touch with the hospital for that.”

We did what we could, including wound debridement, medications to prevent infection, the insertion of K-wire to maintain the integrity of two of the injured fingers, and a promise to refer him for rehabilitation. However, deep inside, I knew the real struggle would begin once he left the hospital.

As he was wheeled into surgery, I thought about the factories, the workshops, and the countless men like Karim who work in dangerous conditions without safety measures. They have no gloves, no protective gear, and no compensation. They are just men trying to earn their next meal, knowing that one mistake could cost them everything. Yet they go on relentlessly working hard, with their spirits bruised but unbroken, clinging to a fragile dream of survival. A better life and a brighter tomorrow hung before them like the moon—visible, shimmering, but forever out of reach, a cruel reminder of what they could see but never hold.

Later that night, as I wrote my notes, I found myself thinking about Karim’s son. Would he have to leave school now? Would he end up in the same factory, facing the same machines?

The reality of this city, of this country, is harsh. As an intern, I had learned one thing: treating wounds is not enough. The real cure lays beyond the hospital walls in the policies that protect workers and in the voices that demand change.

Karim’s case was not the first, and it would not be the last. However, every story and every patient has left a mark. Perhaps, by telling their stories, we can start changing the way things are.

